# Supplementary material for: Substrate Specificity and Structural Modeling of Human Carboxypeptidase Z: A Unique Protease with a Frizzled-Like Domain
Source: Int J Mol Sci. 2020 Nov 18;21(22):8687. doi: 10.3390/ijms21228687 (PMC7698808; doi:10.3390/ijms21228687)
Supplement: Supplementary file 1 [file ijms-21-08687-s001.pdf]

SUPPLEMENTARY MATERIAL

Substrate specificity and structural modeling of human Carboxypeptidase Z: A unique protease with a Frizzled-like domain

Javier Garcia-Pardo[1], Sebastian Tanco[1,2], Maria C. Garcia-Guerrero[1], Sayani Dasgupta[3], Francesc X. Aviles[1], Julia Lorenzo[1*] and Lloyd D. Fricker[3*]

[1]Institut de Biotecnologia i Biomedicina and Departament de Bioquimica i Biologia Molecular, Universitat Autònoma de Barcelona, 08193 Bellaterra, Barcelona, Spain.

[2]BiosenSource BV. B-1800 Vilvoorde,, Belgium

[3]Department of Molecular Pharmacology, Albert Einstein College of Medicine, Bronx, New York 10461, USA.

*To whom correspondence should be addressed: Tel.: +34-935868936; Fax: +34-935812011; E-mail: Julia.Lorenzo@uab.es

*To whom correspondence should be addressed: Tel.: +1-718-430-4225; Fax: +1-718-430-8922; E-mail: Lloyd.Fricker@einstein.yu.edu.

Supplementary Tables

Table S1. Non-substrates of CPZ identified using HEK293T peptides

Precursor	Sequence	Z	T	Obs M	Theor M	ppm	Ratio CPZ / No enzyme			
							100 nM	10 nM	1 nM	0.1 nM
Eukaryotic translation initiation factor 4H	ATPLNQVANPNSAIFGGARPRE EVVQKEQE	4	2	3248.69	3248.65	12	0.83	1.07	1.07	1.07
Acidic nuclear phosphoprotein pp32	STIEPLKK	3	3	914.53	914.54	-20	0.85	0.90	1.15	1.07
Elongation factor 1 beta	GFGDLKSPAGLQV	2	2	1287.68	1287.68	0	0.87	0.87	1.00	0.95
Protein SET (Phosphatase 2A inhibitor I2PP2A) - isoform 2	Ac-SAPAAKVSKKEL	2	3	1269.72	1269.73	-9	0.90	0.93	0.95	0.96
RNA binding motif protein 3	Ac-SSEEGKLFVGGLNF	2	1	1524.77	1524.75	15	0.90	1.00	0.85	0.85
Vimentin	LIKTVETRDGQVINETSQ	3	2	2030.07	2030.04	2	0.90	0.95	0.90	0.95
FK506 Binding Protein	GVQVETISPGDGRTFPKRGQ	4	2	2128.12	2128.12	9	0.94	1.00	1.05	0.91
Ubiquitin-60S ribosomal protein L40	IIEPSLRQL	2	1	1067.64	1067.64	9	0.94	0.90	0.88	0.81
40S Ribosomal protein S28	Ac-MoxDTSRVQPIKLA	2	1	1415.76	1415.75	6	0.94	1.04	1.04	1.19
Complement component 1 Q subcomponent-binding protein, mitochondrial	ADRGVDNTFADELVEL	2	1	1762.86	1762.84	14	0.94	1.06	1.29	1.06
Peptidylprolyl isomerase A	ADKVPKTAENFRAL	4	3	1558.84	1558.850	-7	0.96	0.99	1.02	1.11
Heat shock 10kDa protein 1 (chaperonin 10)	GGIMLPEKSQGKVLQA	3	3	1654.89	1654.90	-9	0.97	0.97	1.07	0.50
Nucleophosmin	ASIEKGGSLPKVEA	3	3	1384.75	1384.76	-5	0.99	0.99	0.95	0.97
40S Ribosomal protein S21	KADGIVSK	3	3	816.44	816.47	-33	1.00	0.94	1.09	1.12
40S Ribosomal protein S28	Ac-MoxDTSRVQPIKL	2	1	1344.72	1344.71	5	1.00	1.07	1.07	1.19
Heat shock 10kDa protein 1 (chaperonin 10)	GGIMLPEKSQGKVL	3	3	1455.79	1455.81	-14	1.00	0.98	1.05	1.00
Heterogeneous nuclear ribonucleoprotein D0	FGGFGEVESIEL	2	1	1282.61	1282.61	2	1.00	1.00	1.13	0.94

Heat shock 10kDa protein 1 (chaperonin 10)	GSGSKGKGGEIQPVSV	3	3	1485.78	1485.78	-2	1.03	0.93	1.13	0.95
Peptidylprolyl isomerase A	ELFADKVPKTA	3	3	1217.65	1217.67	-16	1.05	1.00	1.16	1.05
Cytochrome c oxidase subunit 5a	GISTPEELGLDKV	2	2	1356.72	1356.71	3	1.06	1.00	1.09	1.09
Nucleophosmin	GGFEITPPVVL	2	1	1127.63	1127.62	4	1.06	1.06	1.13	1.06
Peptidylprolyl isomerase A	VNPTVFFDI	2	1	1050.54	1050.54	5	1.07	1.07	0.93	1.07
FK506 Binding Protein	VFDVELL	2	1	833.46	833.45	3	1.07	1.00	1.07	1.14
Heat shock 10kDa protein 1 (chaperonin 10)	TVVAVGSGSKGKGGEIQPVSV	4	3	1955.07	1955.07	1	1.08	1.10	1.14	1.17
FK506 Binding Protein	VFDVELLKLE	2	2	1203.68	1203.68	2	1.08	1.07	1.11	1.08
40S Ribosomal protein S29	AKDIGFIKLD	3	3	1118.61	1118.63	-18	1.08	0.93	1.03	0.98
Cathepsin D	GPIPEVLK	2	2	851.51	851.51	-4	1.08	1.00	1.00	0.83
Protein SET (Phosphatase 2A inhibitor I2PP2A) - isoform 1,2, or 3	SELIAKI	2	2	772.46	772.47	-17	1.11	1.07	1.15	1.11
Heterogeneous nuclear ribonucleoprotein A/B isoform 1, 2 or 3	FGEFGEIEAIEL	2	1	1352.67	1352.65	15	1.11	1.00	1.11	1.06
Heat shock 10kDa protein 1 (chaperonin 10)	VGSGSKGKGGEIQPVSV	3	3	1584.84	1584.85	-3	1.12	1.10	1.13	1.18
Elongation factor 1 beta	GFGDLKSPAGL	2	2	1060.54	1060.56	-10	1.13	1.06	1.06	1.03
CD99 antigen	AEPAVQRTLLEK	3	2	1353.76	1353.76	1	1.13	1.08	1.11	0.91
Nucleophosmin	EKGGSLPKVEA	3	3	1113.57	1113.60	-26	1.13	1.07	1.24	1.00
Peptidylprolyl isomerase A	ELFADKVPKTAENFRAL	4	3	1948.03	1948.04	-4	1.13	0.93	1.03	1.20
60S acidic ribosomal protein P2 polymorphism	DGKNIEDVIAQGIGKL	3	3	1668.88	1668.91	-15	1.14	1.05	1.19	1.09
Superoxide dismutase 1	KGDGPVQGIINF	2	2	1243.65	1243.66	-6	1.14	1.06	1.14	0.94

40S Ribosomal protein S21	KADGIVSKNF	3	3	1077.57	1077.58	-13	1.14	1.03	1.08	1.08
40S Ribosomal protein S21	AKADGIVSKNF	2	3	1148.61	1148.62	-9	1.15	0.86	1.15	0.98
Heterogeneous nuclear ribonucleoprotein D-like	KDAASVDKVLEL	3	3	1286.69	1286.71	-12	1.15	1.09	1.15	1.09
Triosephosphate isomerase 1	LDPKIAVA	2	2	825.48	825.50	-21	1.15	1.06	1.06	1.00
Heat shock 10kDa protein 1 (chaperonin 10)	LPLFDRVLVE	2	1	1199.70	1199.69	9	1.15	1.19	1.19	1.03
60S acidic ribosomal protein P2	VGIEADDDRLNKV	3	2	1442.74	1442.74	0	1.16	1.02	1.05	1.05
Triosephosphate isomerase 1	SLGELIGTLNA	2	1	1086.60	1086.59	3	1.16	1.16	1.19	1.09
40S Ribosomal protein S21	ADGIVSKNF	2	2	949.48	949.48	-6	1.17	1.10	1.10	1.04
60S Ribosomal protein L31	KNLQTVNVDEN	2	2	1272.64	1272.63	4	1.17	0.97	1.10	0.91
Nucleophosmin	TPKTPKGPSSVEDIKA	4	4	1653.87	1653.89	-12	1.17	1.08	1.17	1.04
Heterogeneous nuclear ribonucleoprotein D-like	ASVDKVLEL	2	2	972.54	972.55	-8	1.17	1.06	1.14	1.07
40S Ribosomal protein S21	AKADGIVSKNF	3	3	1148.60	1148.62	-17	1.18	1.06	1.06	0.94
Elongation factor 1 beta	GFGDLKSPAGLQVL	2	2	1400.76	1400.77	-6	1.18	0.94	0.94	0.82
Nucleophosmin	GGSLPKVEA	2	2	856.46	856.47	-10	1.18	0.99	1.13	0.99
FK506 Binding Protein	VELLKLE	2	2	842.50	842.51	-14	1.19	1.00	1.14	1.04
Complement component 1 Q subcomponent-binding protein, mitochondrial	DRGVDNTFADELVELSTA	2	1	1950.95	1950.92	17	1.19	1.13	0.88	1.13

Non-substrates; peptides no affected (with a decrease ≤20% or an increase ≤120% by the highest concentration of enzyme). See Table 2 for abbreviation definitions.

Table S2. Non-substrates of CPZ identified using the tryptic peptide library

Protein precursor	Sequence	Z	T	Obs M	Theor M	ppm	Ratio CPZ / No enzyme		
							100 nM	10 nM	1 nM
α-Hemoglobin	VDPVNFK	2	2	817.42	817.43	-14	0.82	0.76	0.91
α-Hemoglobin	LRVDPVNFK	3	2	1086.62	1086.62	-3	0.83	0.80	0.75
Thyroglobulin	ILNDAQTK	2	2	901.48	901.49	-11	0.85	0.82	0.88
Thyroglobulin	VTLAADR	2	1	744.42	744.41	12	0.86	0.86	0.97
Thyroglobulin	ETFLEK	2	2	765.38	765.39	-8	0.87	0.90	1.00
Thyroglobulin	SALGEPKK	2	1	828.48	828.47	7	0.89	0.86	0.97
Thyroglobulin	KFEKLPESK	4	4	1104.59	1104.62	-27	0.89	0.90	0.97
Bovine serum albumin	LVNELTEFAK	2	2	1162.62	1162.62	-4	0.89	0.89	0.92
Thyroglobulin	IDVALR	2	1	685.42	685.41	12	0.90	0.90	0.93
Thyroglobulin	QAGVQAEPSPK	2	2	1110.57	1110.57	0	0.91	0.83	1.00
Thyroglobulin	RSLLLAPEEGPVSQR	3	1	1650.93	1650.91	13	0.93	0.93	1.00
Thyroglobulin	ALADLAKP	2	2	797.45	797.46	-17	0.93	0.83	0.90
Thyroglobulin	AVKQFEESQGR	2	2	1277.64	1277.64	1	0.94	0.91	0.97
Thyroglobulin	ELSVLLPNR	2	1	1039.61	1039.60	8	0.94	1.00	1.00
Thyroglobulin	LTDEELAFPPLSPSR	3	1	1670.86	1670.85	7	0.95	1.08	1.14
Bovine serum albumin	DDSPDLPK	2	2	885.39	885.41	-19	0.95	0.92	0.97
α-Hemoglobin	VLSPADKTNVK	3	3	1170.65	1170.66	-5	0.96	0.91	0.88
Bovine serum albumin	LVTDLTK	2	2	788.45	788.46	-7	0.97	0.92	0.90
Thyroglobulin	LGGQEIR	2	1	771.43	771.42	12	0.98	0.95	0.96
Thyroglobulin	SLSLK	2	2	546.33	546.34	-24	1.00	0.83	0.83
Thyroglobulin	KVVLQDR	2	2	856.51	856.51	0	1.00	0.97	0.97
Thyroglobulin	FVAPESLK	2	2	889.48	889.49	-6	1.00	0.93	0.93
Thyroglobulin	ALADLAKPL	2	2	910.55	910.55	1	1.00	0.91	0.94
Thyroglobulin	ASGLGAAAGQR	2	1	957.50	957.50	2	1.00	0.90	0.97
Thyroglobulin	LVTLAESPR	2	1	984.57	984.56	8	1.00	0.93	0.86

Thyroglobulin	LNSNPASEAPK	2	2	1126.56	1126.56	1	1.00	0.83	0.86
Thyroglobulin	KGQEFTITGQK	2	3	1235.63	1235.65	-13	1.00	0.87	0.89
Thyroglobulin	VVLQDR	2	1	728.42	728.42	-1	1.01	0.94	1.05
Thyroglobulin	SLLLAPEEGPVSQR	3	1	1494.81	1494.80	7	1.02	0.86	1.09
Bovine serum albumin	DAIPENLPPLTADFAEDK	3	2	1954.97	1954.95	8	1.03	0.90	1.05
Bovine serum albumin	LVVSTQTALA	2	1	1001.58	1001.58	0	1.03	1.06	1.07
α-Hemoglobin	Ac-VLSPADKTNVK	2	2	1212.67	1212.67	1	1.03	1.08	1.02
Thyroglobulin	QQAAALAK	2	2	799.44	799.46	-20	1.03	0.87	0.93
Bovine serum albumin	AEFVEVTK	2	2	921.47	921.48	-7	1.05	0.97	1.01
Trypsin	VATVSLPR	2	1	841.51	841.50	12	1.06	0.91	1.03
Thyroglobulin	FAATSFR	2	1	798.41	798.40	8	1.07	1.00	1.03
Thyroglobulin	GQEIPGTR	2	1	856.45	856.44	11	1.07	1.00	1.07
Thyroglobulin	LQQNLFGGR	2	1	1031.54	1031.55	-9	1.07	1.00	0.97
Thyroglobulin	FLQGDR	2	1	734.37	734.37	6	1.08	0.94	1.00
Thyroglobulin	RLVTLAESPR	3	1	1140.67	1140.66	11	1.09	0.97	0.98
Thyroglobulin	LTDEELAFPPLSPSRETFLEK	3	2	2418.25	2418.23	9	1.09	0.97	1.00
β-Hemoglobin	VNVDEVGGEALGR	2	1	1313.67	1313.66	10	1.17	1.00	1.17
Thyroglobulin	VDLLIGSSQDDGLINR	2	1	1713.91	1713.89	14	1.18	1.09	1.18

[1]Fragment originated from trypsin autolysis; Non-substrates; peptides no affected (with a decrease ≤20% or an increase ≤120% by the highest concentration of enzyme). See Table 2 for abbreviation definitions.

Supplementary Figures

Figure S1

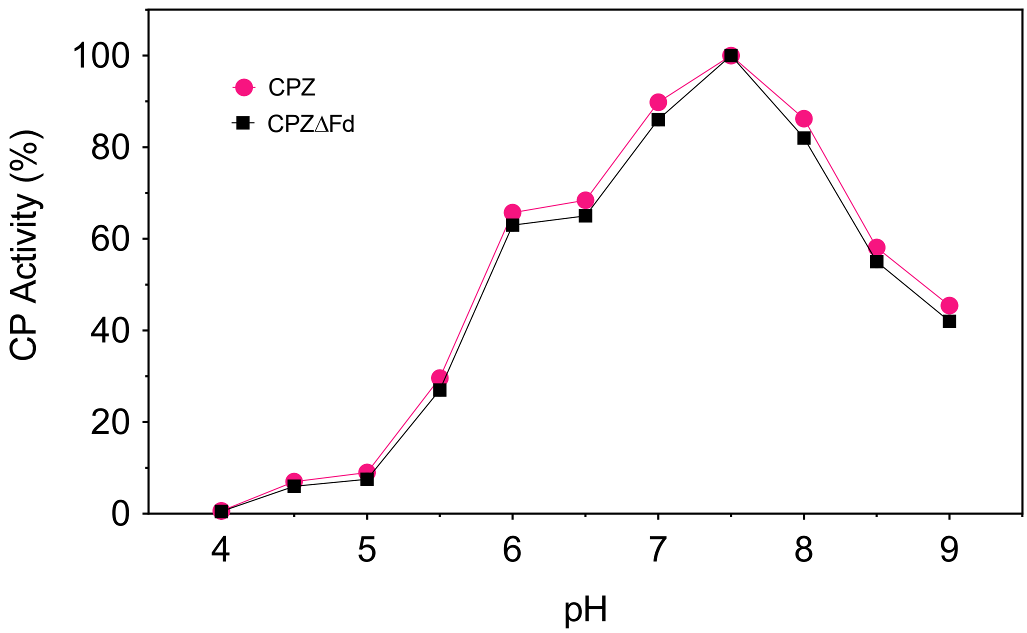

Figure S1. Effect of pH on the carboxypeptidase activity of recombinant purified CPZ proteins. Effect of pH on CPZ and CPZΔFz activity using 200 µM dansyl-Phe-Ala-Arg in a Tris-acetate buffer at the indicated pH for 60 min at 37ºC, as described in the experimental section. Carboxypeptidase activity was represented to the maximal activity at optimal pH and represents the average of three independent measurements (with less than 10% variation

Figure S2

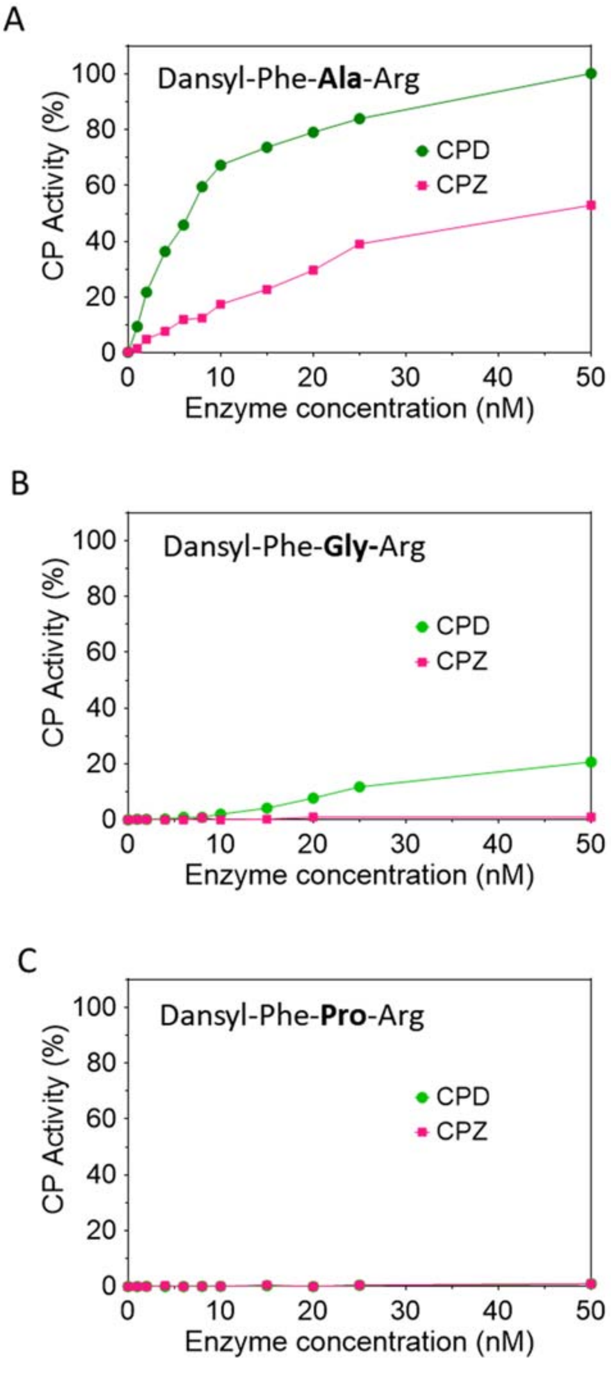

Figure S2. Relative amount of product formed by three different dansylated tripeptides incubated with various amounts of purified CPZ and CPD (as control enzyme). Reactions containing 200 µM of (A) dansyl-Phe-Ala-Arg, (B) dansyl-Phe-Gly-Arg, or (C) dansyl-Phe-Pro-Arg were incubated with different amounts of CPZ (red line) or CPD (green line) in 100 mM Tris-acetate, pH 7.5 buffer (for CPZ) or 100 mM Tris-acetate, pH 6.9, 150 mM NaCl buffer (for CPD), for 60 min at 37ºC. Samples were analyzed as described in the experimental section.
